# Multicenter Study on Breast Cancer in the Geriatric Population: Insights for Effective Treatment Strategies

**DOI:** 10.7759/cureus.57253

**Published:** 2024-03-30

**Authors:** Sule Karabulut Gul, Huseyin Tepetam, Berrin Benli Yavuz, Ozge Kandemir Gursel, Ayşe Altinok, Pelin Altinok, Ahmet Fatih Oruc, Duygu Akincioglu, Raghad Al Shomali, Omar Alomari, Mehmet Alper Kaya

**Affiliations:** 1 Radiation Oncology, Kartal Dr. Lutfi Kirdar Training and Research Hospital, Istanbul, TUR; 2 Radiation Oncology, Meram Medical Faculty - Necmettin Erbakan University, Konya, TUR; 3 Radiation Oncology, Prof. Dr. Cemil Tascioglu City Hospital, Istanbul, TUR; 4 Radiation Oncology, Medical Park Hospital, Istanbul, TUR; 5 Radiation Oncology, Umraniye Training and Research Hospital, Istanbul, TUR; 6 Radiation Oncology, Istanbul Oncology Hospital, Istanbul, TUR; 7 Radiation Oncology, Hamidiye International School of Medicine, University of Health Sciences, Istanbul, TUR

**Keywords:** multicenter study, treatment approaches, multicenter analysis, elderly patients, breast cancer

## Abstract

Objective: Breast cancer is common among women aged 65 and over. There is a significant lack of evidence regarding the treatment of breast cancer in patients in this age group due to the rare inclusion of these patients in clinical studies. However, it is known that survival in elderly patients with breast cancer is significantly reduced in those not receiving standard therapy. Several factors, including patients’ comorbidities, performance status, life expectancy, and tumor pathological and molecular characteristics, can affect the outcomes of treatment. In this study, we aimed to update the knowledge in this field by assessing these factors among the geriatric population in our multicenter dataset.

Methods: This retrospective study analyzed data from 335 breast cancer patients aged 65 and over who received adjuvant radiotherapy at five oncology centers (Kartal Dr. Lutfi Kirdar Training and Research Hospital, Istanbul, Meram Medical Faculty - Necmettin Erbakan University, Konya, Prof. Dr. Cemil Tascioglu City Hospital, Istanbul, Umraniye Training and Research Hospital, Istanbul, and Istanbul Oncology Hospital, Istanbul) between May 2010 and September 2022. Demographic, clinical, and pathological data were collected, including age, gender, clinical symptoms, tumor characteristics, treatment approaches, and outcomes. Statistical analyses, including descriptive statistics, Kaplan-Meier analysis, log-rank test, and Cox regression analysis, were performed using IBM SPSS Statistics for Windows, Version 22 (Released 2013; IBM Corp., Armonk, New York, United States), with a significance level of p < 0.05.

Results: The tumor characteristics and survival time of 335 breast cancer patients were examined. In the results, performance status, T stage, and perineural invasion were found to be factors affecting the survival of elderly breast cancer patients. In multivariate analysis, it was seen that performance status played an important role as an independent prognostic factor.

Conclusion: The treatment of breast cancer in the geriatric age group necessitates a personalized approach, taking into account the patient's overall health status, life expectancy, and comorbidities.

## Introduction

Breast cancer is the most common cancer diagnosed among women worldwide [1]. More than half of the patients are above the age of 60 years and the prevalence is increasing in older adults [2]. Older women have a higher risk of mortality and treatment-related morbidity when compared to younger women [3]. This could be explained by advanced presentation, delayed diagnosis, organ function decline, and the presence of comorbidity [4].

However, due to the heterogeneous nature of the disease and the paucity of evidence specific to older persons, managing breast cancer in this population is challenging [5]. The under-representation and exclusion of elderly persons from clinical studies led to major gaps in evidence regarding the evaluation and treatment of this population [6,7]. The few studies that focused specifically on breast cancer in elderly women provided some details about the clinicopathological features of the disease in this group and showed that it differs uniquely from the features of the disease in the younger population [8,9].

Due to unjustifiable fears of advanced age and associated comorbidities, chemotherapy, radiotherapy, and even surgical resection of the tumor are frequently delayed or avoided in older patients with breast cancer [10-12]. Such undertreatment may have a deleterious effect on prognosis and treatment outcomes [13]. In order to prevent unnecessary undertreatment or the exposure of patients to intolerable toxicities, proper geriatric assessment is crucial [2]. This includes but is not limited to evaluations such as Activities of Daily Living (ADL), Montreal Cognitive Assessment (MoCA), Geriatric Depression Scale (GDS), Mini Nutritional Assessment (MNA), and Timed Get Up and Go (TGUG). Recent recommendations, such as the de-escalation of surgical axillary staging in patients over 70, as indicated by Grossi et al. (2023), highlight the importance of personalized treatment approaches and careful consideration of the risks and benefits in this population [14]. Furthermore, recent studies, including LUMINA, CALGB 9343, and PRIME II, have emphasized alternative treatment approaches for older patients with breast cancer. These studies provide insights into the efficacy and safety of omitting radiotherapy in selected older patients, contributing to the discussion on individualized treatment decisions [15-17].

Assessment of several factors, including patients’ comorbidities, performance status, life expectancy, and tumor pathological and molecular characteristics, should be carefully taken into account in decision-making [2]. Considering the limited applicability of the evidence produced in younger individuals, more studies addressing these factors in geriatric patients and their effect on mortality, treatment-related morbidity, and quality of life are needed for the optimization of the treatment guidelines in this group.

Despite the considerable progress in breast cancer diagnosis and treatment, there remains a paucity of research concerning the geriatric population. As the global life expectancy increases, so too does the prevalence of geriatric breast cancer patients, who present a distinct set of challenges and considerations compared to younger patients. The current lack of research regarding this population limits our understanding of how to effectively treat and manage breast cancer in this age group. Therefore, this study aims to fill this knowledge gap and provide valuable insights into improving the care of geriatric breast cancer patients.

The objective of this study is to assess the patient, tumor, and treatment characteristics of breast cancer among the geriatric population in our multicenter dataset. By examining the social, psychological, and economic impact of the treatment process on patients, we can adopt a more comprehensive and effective approach to treating breast cancer in elderly patients [18]. Through a thorough analysis of this information, we aim to identify any patterns or trends that can inform more effective treatment strategies for this population.

## Materials and methods

Study design and patient population

This retrospective study analyzed data from 335 patients aged 65 and over who were diagnosed with histo-pathologically confirmed breast cancer and received adjuvant radiotherapy at five different oncology centers between May 2010 and September 2022. The study recorded the demographic, clinical, and pathological characteristics of the patients, as well as their treatment approaches and outcomes. 

The study collected demographic data such as age, gender, education level, and socioeconomic status. Clinical characteristics, including patients' presenting symptoms, comorbidities, and performance status, were also taken into account. Pathological characteristics were analyzed and covered important cancer properties such as tumor type, stage, size, histological grade, lymph node status, hormonal receptor status, HER2 status, and other biomarkers. The collected data were used for both prognostic and predictive evaluations of the patients. Treatment approaches and outcomes examined the patients' exposure to various treatment methods, including surgical, neoadjuvant, and adjuvant chemotherapy, radiotherapy, and hormonal therapy. Additionally, the study assessed the post-treatment complications, side effects, and quality of life of patients. The psychosocial support and educational needs of patients during the treatment process were also taken into consideration.

Statistical analyses

All statistical analyses were performed using IBM SPSS Statistics for Windows, Version 22 (Released 2013; IBM Corp., Armonk, New York, United States). Descriptive statistics were calculated to examine the basic characteristics of the data, including means, medians, standard deviations, percentage values, frequencies of demographic features, clinical-pathological findings, and treatment methods of the patients. To evaluate survival rates and compare them between different groups, the Kaplan-Meier analysis was utilized. The log-rank test was then used to determine the statistical significance of differences between the groups. Additionally, Cox regression analysis was conducted for multivariate analysis. A p-value of less than 0.05 was considered statistically significant for all analyses. This value was utilized to evaluate the reliability of the statistical findings obtained from the study.

## Results

In this study, tumor characteristics and survival time of 335 breast cancer patients were examined. The mean age of the patients participating in the study was 70 (65-88), and the follow-up period was 40.40 (4.27-196.44) months. 58.5% of the patients also had an accompanying chronic disease. The most common histology type was invasive ductal carcinoma (57%). The tumor was located on the left side in 170 patients. 88.3%, 74.5%, and 16% of the patients had estrogen receptor, progesterone receptor, and c-erbB-2 positivity respectively. Luminal B subtype was the most common with 66% of the patients having this cancer type. Grade 3 toxicity was seen in only ten patients, while 13 patients discontinued treatment due to side effects. Treatment modalities and other treatment characteristics are summarized in Table 1 and Table 2.

**Table 1:** Patients’ characteristics. *ECE: Extracapsular Extension

Characteristics	n (%)
ECOG	
0	1 (3)
1	289 (86.3)
2	34 (10.1)
3	2 (0.6)
T stage	
T 1-2	298 (89)
T 3-4	35 (10.4)
Not known	2 (0.6)
N stage	
N 0-1	264 (78.8)
N 2-3	71 (21.2)
ECE*	
No	204 (60.9)
Yes	95 (28.4)
Not known	36 (10.7)
Grade	
1	37 (11)
2	205 (61.2)
3	88 (26.3)
Not known	5 (1.5)

**Table 2:** Treatment characteristics. *BCS: Breast Conserving Surgery; MRM: Modified Radical Mastectomy; SNLB: Sentinel Lymph Node Biopsy; ALND: Axillary Lymph Node Dissection.

Characteristics	n (%)	
Breast Surgery		
BCS*	170 (50.7)	
MRM*	156 (46.6)	
MRM + reconstruction	1 (0.3)	
Excisional biopsy	8 (2.4)	
Axillary Surgery		
No	6 (1.8)	
SLNB*	131 (39.1)	
ALND*	156 (46.6)	
SLNB+ALND	42 (12.5)	
Chemotherapy		
No		91 (27.2)
Yes		244 (72.8)
Concurrent hormone therapy		
No	134 (40)	
Yes	185 (55.2)	
Not known	16 (4.8)	
Radiotherapy dose		
Conventional	228 (68.1)	
Hypofractionated	106 (31.6)	
Not known	1 (0.3)	

**Figure 1:**
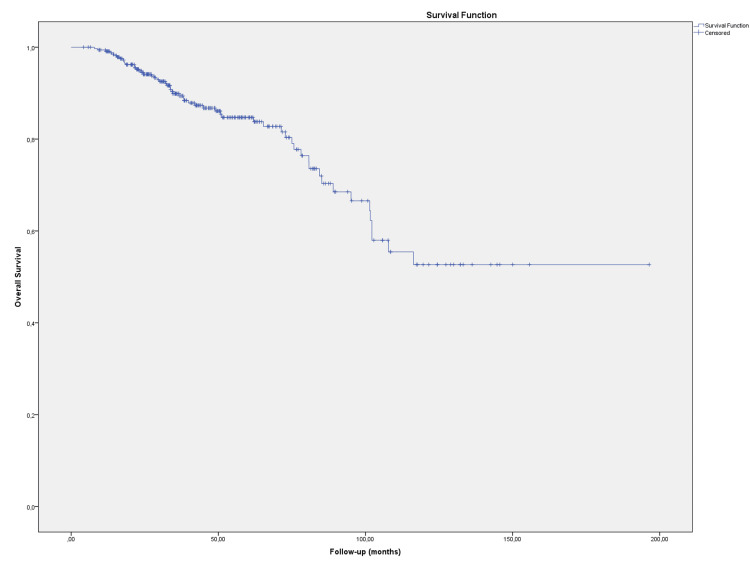
Overall survival rates of patients. Kaplan-Meier survival curves show the percentage of patients who survived at 2, 5, and 10 years after treatment. The numbers above the curves indicate the survival rates at each time point.

The overall survival (OS) rates of the patients at two, five, and ten years were 94.8%, 84.7%, and 52.7%, respectively (Figure 1).

At the end of the follow-up period, 279 patients (83.3%) were alive. According to univariate analysis results, performance status (ECOG 0-1/2-3; p=0.001), T stage (T1-2/T3-4; p=0.025), and perineural invasion (PNI) (absent/present; p =0.045) were found to be important factors affecting the OS. ​​​​​In multivariate analysis, only performance status (P=0.002, HR: 2.995, 95% CI: 1.508-5.951) was considered an independent prognostic factor for OS. In conclusion, performance status, T stage, and perineural invasion were found to be factors affecting the survival of elderly breast cancer patients. In multivariate analysis, it was seen that performance status played an important role as an independent prognostic factor.

## Discussion

Breast cancer tends to present with less aggressive features as age increases, and with age, estrogen receptor (ER) expression increases while HER2 expression decreases [19]. The natural progression, diagnosis, and treatment approaches of breast cancer in the geriatric age group are similar to those in young women with breast cancer. Our study's results align with previous research on the distribution of pathological features in elderly breast cancer patients. In particular, Bastiaannet et al. found that lower-grade tumors and higher rates of hormone receptor expression were more prevalent among this age group [20]. These findings are consistent with our own, suggesting a general trend in the type of breast cancer that occurs in older individuals. 

It is noteworthy that the prevalence of lower-risk subtypes such as luminal A and luminal B was higher among the majority of elderly patients studied [21]. This situation has the potential to offer a better prognosis and appropriate treatment options for elderly patients. Our study also supports this with the observation of a higher prevalence of Luminal A and B subtypes among the patients. This finding suggests that there may be a greater likelihood of treating elderly patients with less aggressive, hormone receptor-positive tumors, resulting in better outcomes.

In our study, we found that the stage distribution and T stages of breast cancer patients were generally similar to those of other studies in the literature. This is consistent with a study conducted by Yancik et al., which reported that elderly patients have a higher incidence of breast cancer compared to younger patients, particularly with regard to lower stage tumors [18].

It is important to note that early detection of breast cancer is crucial for successful treatment outcomes, and the stage of cancer at the time of diagnosis plays a critical role in determining the appropriate treatment options. Therefore, our findings highlight the importance of regular breast cancer screenings for all age groups, especially for elderly patients who may be at a higher risk for breast cancer. The findings suggest that early-stage tumors are more common in elderly patients and are associated with a better prognosis. 

Our study also showed a higher proportion of patients with lower T stages, consistent with other studies in the literature [22]. These results have significant implications for the treatment and management of breast cancer in older patients, as early detection and appropriate intervention could lead to better outcomes. We also recognize the rationale for de-escalation of treatment in this population, where less aggressive approaches may yield equal outcomes. Moreover, further research is needed to identify potential factors that contribute to the higher prevalence of early-stage tumors in this population, such as differences in screening practices or biological mechanisms underlying age-related changes in tumor development.

The incidence rates of right and left breast cancer observed in our study are similar to those reported in other studies in the literature. Overall, no significant difference was observed in the rates of right and left breast cancer [23]. This finding suggests that breast cancer can occur in both breasts at similar rates, supporting the validity of treatment approaches for both sides. It is important to note that the similarity in incidence rates of right and left breast cancer may have implications for surgical decisions and follow-up care for breast cancer patients. Further research is needed to explore the potential impact of these findings on clinical practice. 

Our study reveals that the surgical methods applied to geriatric breast cancer patients are similar to those in other studies in the literature. This is confirmed by a study conducted by Alderman et al., which demonstrates that surgical approaches in elderly patients are similar to those in young patients in terms of quality of life, survival, and side effects [24]. The similarity of the surgical methods used in elderly and young patients implies that age alone should not be a decisive factor in determining the surgical approach. Rather, each patient should be evaluated individually, and the treatment plan should be tailored to their specific needs and circumstances. Studies show that in general, older patients with early-stage tumors are treated with less invasive methods such as lumpectomy or partial mastectomy instead of mastectomy [25]. This supports the use of less invasive methods in the treatment of breast cancer in older patients to preserve their quality of life. 

It is essential to consider the age, general health status, and treatment preferences of older patients when developing treatment plans to ensure that the chosen methods will provide optimal outcomes with minimal side effects. Additionally, studies have shown that the use of multidisciplinary approaches, including geriatric assessments and supportive care, can improve treatment decision-making and overall outcomes for older breast cancer patients.

In our study, we examined the toxicity of breast cancer treatments in the geriatric age group, and it was found that serious side effects were not encountered. This is consistent with other studies in the literature. For instance, Whelan et al. conducted a study in which acute and late toxicities were observed to be low in geriatric patients who received hypofractionated radiotherapy [26]. These results suggest that breast cancer treatments can be well-tolerated in the geriatric population with minimal side effects. In addition, a study conducted by Wildiers et al. reported that appropriate treatment management and dose adjustments for elderly patients with breast cancer significantly reduced the risk of toxicity compared to younger patients [22]. This finding is of great importance as it highlights the need for individualized treatment approaches for different age groups. Moreover, the study suggests that with proper attention to dose adjustments and monitoring, elderly patients can receive similar cancer treatments as younger patients without experiencing higher rates of toxicity. This information can be particularly reassuring to elderly breast cancer patients and their caregivers, who may be concerned about the potential risks associated with cancer treatments.

Hypofractionated radiotherapy is an important treatment option, especially for elderly patients, as it improves quality of life and treatment duration while reducing toxicity. Our study also found that this method was successfully applied to appropriate patients. The Fast Forward study (Brunt et al., 2020) highlights the benefits of a one-week radiotherapy regimen, ideal for elderly patients. This condensed approach ensures convenience without compromising treatment effectiveness [27]. During treatment, life-threatening or interruption-requiring side effects were not observed by considering the patient's performance. In our study, we carefully planned treatment approaches by taking into consideration the performance of each patient. This individualized approach allowed us to minimize the risk of life-threatening or treatment-interrupting side effects. During the course of treatment, we monitored patients closely and found no significant adverse events that would require a pause or cessation of treatment. By customizing our treatment strategies based on the patient's unique needs and abilities, we were able to provide a safe and effective treatment course that maximized the chances of success while minimizing the risk of harm. Our findings highlight the importance of personalized care in improving patient outcomes and quality of life.

In today's medical landscape, hypofractionated radiation therapy regimens are increasingly being considered as a viable treatment option for patients. However, it is crucial to evaluate each patient on an individual basis, taking into account any comorbidities or fragilities that may be present. While it is important to exercise caution in administering hypofractionated radiation therapy, avoiding its use altogether in appropriate patients could potentially lead to missed opportunities for effective treatment. Therefore, careful consideration and evaluation of each patient's unique situation is necessary to determine the best course of treatment.

Limitations of the study

This study has certain limitations, such as its retrospective nature that may have led to the use of outdated risk scoring methods and potential mismatch with current clinical practices. Furthermore, it is unclear whether our findings are generalizable to elderly patients in different geographic regions and healthcare systems. To address these limitations, larger and multicenter studies with participants from diverse countries are necessary to enhance the generalizability of the results. 

## Conclusions

In this study, we have provided valuable insights into the complexities of breast cancer management in the geriatric population. Our comprehensive analysis of 335 elderly breast cancer patients revealed distinct tumor characteristics, treatment patterns, and outcomes, highlighting the importance of tailored approaches in their care. Notably, the predominance of less aggressive tumor features and hormone receptor-positive subtypes suggests promising prognoses and opportunities for less intensive yet effective treatment strategies.

Crucially, our findings underscore the pivotal role of performance status in predicting outcomes, emphasizing the necessity of comprehensive geriatric assessments in treatment decision-making. Furthermore, our study emphasizes the significance of early detection through regular screenings, the well-tolerated nature of treatments, and the benefits of incorporating hypofractionated radiotherapy. While contributing significantly to the understanding of geriatric breast cancer, the scarcity of studies in Turkey underscores the need for larger sample sizes and extended follow-up periods to address population-specific challenges comprehensively. Ultimately, our research advocates for personalized, multidisciplinary approaches to optimize care for elderly breast cancer patients, urging ongoing efforts to enhance their quality of life through tailored interventions.
